# Microbes in Tumoral In Situ Tissues and in Tumorigenesis

**DOI:** 10.3389/fcimb.2020.572570

**Published:** 2020-11-24

**Authors:** Xue Feng, Lu Han, Sijia Ma, Lanbo Zhao, Lei Wang, Kailu Zhang, Panyue Yin, Lin Guo, Wei Jing, Qiling Li

**Affiliations:** Department of Obstetrics and Gynecology, The First Affiliated Hospital of Xi’an Jiaotong University, Xi’an, China

**Keywords:** microbes, cancer, tumors, pathogenesis, relationships

## Abstract

Cancerous tumors are severe diseases affecting human health that have a complicated etiology and pathogenesis. Microbes have been considered to be related to the development and progression of numerous tumors through various pathogenic mechanisms in recent studies. Bacteria, which have so far remained the most studied microbes worldwide, have four major possible special pathogenic mechanisms (modulation of inflammation, immunity, DNA damage, and metabolism) that are related to carcinogenesis. This review aims to macroscopically summarize and verify the relationships between microbes and tumoral *in situ* tissues from cancers of four major different systems (urinary, respiratory, digestive, and reproductive); the abovementioned four microbial pathogenic mechanisms, as well as some synergistic pathogenic mechanisms, are also discussed. Once the etiologic role of microbes and their precise pathogenic mechanisms in carcinogenesis are known, the early prevention, diagnosis, and treatment of cancers would progress significantly.

## Introduction

Malignant tumors are the second leading cause of death for men and the third leading cause of death for women (Whitlock et al., 2015). The most common malignant tumors are related to lung cancer, colorectal cancer, breast cancer, and prostate cancer in high-income countries and stomach cancer, liver cancer, esophageal cancer, and cervical cancer in low-income countries. Tumors are complex entities formed by cancer cells and the tumor stroma *via* multiple and bidirectional interactions to establish dependencies essential for initiating tumorigenesis (Zanconato et al., 2019). The pathogenesis of malignant tumors is correlated with genetic inheritance, environment, dietary habits, radiation, and endocrine disorders (Torre et al., 2016). The factors related to cancer can be divided into two categories: exogenous and endogenous (Whitlock et al., 2015; Torre et al., 2016; Collaborators, U.S.B.o.D et al., 2018; D’Souza et al., 2019; Zanconato et al., 2019). Existing studies have proposed some possible relationships between tumors and microbes in different types of cancers (Nilsson et al., 2006; Guo et al., 2012; Kostic et al., 2012; Garrett, 2015; Mitsuhashi et al., 2015; Audirac-Chalifour et al., 2016; Lee Y. C. et al., 2016; Liu H. X. et al., 2018; Okushin et al., 2018; Aykut et al., 2019; Haruki et al., 2019). Cancers from different systems have unique observable microbes and modes of pathogenesis (**Table 1**). The cancer-related microbes, which play a potential vital role in carcinogenesis, might originate from exogenous infections or microbial dysbiosis. The latter means the composition of microbial community in dysbiotic individuals is significantly different from healthy individuals (Goodwin, 1988; Tan and Wong, 2011; Shanmughapriya et al., 2012; Cavarretta et al., 2017; Mao et al., 2018; Pushalkar et al., 2018; Brüssow, 2020).

**Table 1:** The main mentioned cancers in this review and the usually observable and representative microbes and pathogenesis.

Cancers	Microbes	Possible pathogenic mechanisms
Prostate cancer	*Mycoplasma genitalium*	Infecting and inducing both symptomatic and asymptomatic inflammatory responses in the prostate (Yoon et al., 2012; Caini et al., 2014; Cavarretta et al., 2017); bacterial protein products, such as p37, that exert oncogenic effects (Ketcham et al., 2005; Goodison et al., 2007).
Lung cancer	*Haemophilus inﬂuenzae, Enterobacter* spp., *Escherichia coli, Capnocytophaga* and *Veillonella, Streptococcus viridans, Firmicutes, TM7, Megasphaera, Granulicatella, Abiotrophia, Thermus, Streptococcus viridans, Legionella*, *tuberculosis*	Microbial dysbiosis, genotoxicity and virulence effects, inﬂammation, immune responses, and metabolism (Weitzman and Gordon, 1990; Coussens and Werb, 2002; Ballaz and Mulshine, 2003; Roesler et al., 2012; Liu H. X. et al., 2018); chronic inﬂammation-associated carcinogenesis, with an increase in tumor necrosis factor and excessive and persistent local inflammation at sites of repair and fibrosis (Coussens and Werb, 2002; Ardies, 2003); low immunity (Christopoulos et al., 2014; Khoruts, 2018; Pandey et al., 2019); some metabolic-related signaling pathways, such as amino acid metabolism, carbohydrate metabolism, energy metabolism, and lipid metabolism (Gomes et al., 2019); upregulate the phosphoinositide 3-kinase pathway to participate in regulating cell proliferation, survival, and differentiation (Mendoza et al., 2011; Tsay et al., 2018); reduced signal transduction, increased excretory systems, amino acid metabolism, aldosterone-regulated sodium reabsorption, or amoebiasis pathways (Gomes et al., 2019); the cytokines might induce a cytokine cascade and a proliferation of the lung epithelial cells, the breaks in the chromosomal strands and the accumulation of DNA mutational changes might be eventually activated (Weitzman and Gordon, 1990; Ballaz and Mulshine, 2003); regional tumor peptides and even radiotherapy might lead to a microenvironment deregulation in granulomas (Christopoulos et al., 2014).
Pancreatic cancer	*H. pylori*, *Fusobacterium*, *Lepotrichia*, *Malassezia*	Activate selected toll-like receptors (TLR) in monocytic cells to generate a tolerogenic immune program, TLR2 and TLR5 ligation was demonstrated to induce innate and adaptive immune suppression to promote PDA (Pushalkar et al., 2018); the activation of the mannose-binding lectin–C3 cascade through the C3 complement pathway might cause inflammation induced by the oncogenic Kras, leading to fungal dysbiosis and promoting tumor progression (Aykut et al., 2019).
Colorectal cancer	*Fusobacterium*, *Oribacterium*, *Prevotella*, *Citrobacter rodentium*, *Salmonella enterica, E.coli*, *Pseudomonas aeruginosa, Bacteroides fragilis, Campylobacter* spp.*, E. Faecalis*	Destroy the intestinal barrier, leading to the subsequent induction of pro-inﬂammatory cytokines, such as the reactive oxygen species (ROS), which promoted regeneration and predisposed to tumorigenesis (Kuraishy et al., 2011; Kux and Pitsouli, 2014; Li et al., 2019); *Fusobacterium* could bind to host epithelial Cadherin 1 through the adhesion of FadA and invade epithelial cells from through the E-cadherin/β-catenin signaling to induce inflammation and tumor cell growth in transformed cells (Rubinstein et al., 2013; Wong and Yu, 2019; Guo P. et al., 2020); enhance genomic instability both in two-dimensional and organotypic three-dimensional tissue models (Fearon, 2010); the deficiency in APC is associated with the sustained activation of the DNA damage response and the reduced capacity to repair different types of damage, including DNA breaks and oxidative damages. Infection with genotoxic *Salmonella* was shown to prevent cell cycle arrest in APC-deficient cells (Martin et al., 2019); The cytolethal distending toxin produced by *Escherichia* and *Campylobacter* spp. could induce double-strand DNA break *via* its deoxyribonuclease activity to develop cancer (Cuevas-Ramos et al., 2010; He et al., 2019); Colibactin produced by members of the Enterobacteriaceae family could also induce DNA strand break (Buc et al., 2013); *B. fragilis* toxin (Goodwin et al., 2011) and ROS produced by *E. Faecalis* were both associated with DNA damage and genomic instability *in vitro* (Huycke, 2002; Wang and Huycke, 2007); produce metabolites or genotoxins, such as cytolethal distending toxin and colibactin, some of them can be directly pro-carcinogenic or opportunistic microorganisms in the tumor-associated microenvironment. The cytolethal distending toxin produced by *Escherichia* and *Campylobacter* spp. (Cuevas-Ramos et al., 2010; He et al., 2019), Colibactin produced by members of the Enterobacteriaceae family (Buc et al., 2013), *B. fragilis* toxin (Goodwin et al., 2011), and ROS produced by *E. Faecalis* could induce the DNA strand break which might be associated with tumorigenesis (Huycke, 2002; Wang and Huycke, 2007); activate the pro-carcinogenic signaling pathways and result in molecular changes, ultimately leading to cancer (Wong and Yu, 2019; Zorron Cheng Tao Pu et al., 2019); interfere with signaling pathways to affect several cytokines and growth factors, such as IL-6, IL-2, and tumor necrosis factor, to control the process of regeneration in injured intestinal mucosa (Stavria and Yiorgos, 2013; Karin and Clevers, 2016); induce double-strand DNA breaks *via* its deoxyribonuclease activity to develop cancer (Cuevas-Ramos et al., 2010; He et al., 2019); the exposure of CRC cells to bacterial ﬂagellin increased IL6 and *CCL2/MCP-1* mRNA expression and IL6 excretion. Flagellin was shown to decrease caspase-1 activity and the production of reactive oxygen species to increase cytotoxicity in C26 cells, deteriorate the C2C12-myotubes, and decrease their numbers (Pekkala et al., 2019); the DNA damage was obviously enhanced in the mice colon epithelial cells (2012); *Bacteroides fragilis* promoted the IL-17 induction with early augmentation by pks+ *E. coli* cocolonization (2018); transform the tolerogenic apoptosis of ileal intestinal epithelial cells into immunogenic cell demise, then elicit IL-1β-dependent follicular T helper responses (Roberti et al., 2020); Chronic mucosal formation of IL17A produced by Th 17 cell might alter signaling pathways in colon epithelial cells or induce changes or mutations in DNA structure that facilitated the transformation of colon epithelial cells contributing to carcinogenesis (Hurtado et al., 2018); upregulated the spermine oxidase (SMOX) gene expression in human normal colon epithelial cells, the SMO protein played an important role in the alteration of polyamine metabolism, which catalyzed the oxidation of spermine to spermidine and produced hydrogen peroxide and aldehydes to result in apoptosis, DNA damage, and consequently the development of CRC (Pekkala et al., 2019); the intestinal infection with *Pseudomonas aeruginosa* with a latent oncogenic form of the Ras1 oncogene was found to lead to massive over-proliferation of intestinal cells through activating the c-Jun N-terminal kinase (JNK) pathway as a homeostatic compensatory mechanism to replenish the apoptotic enterocytes (Apidianakis et al., 2009). The Imd–dTab2–dTak1 innate immune pathway was converged with Ras1V12 signaling on JNK pathway activation to induce the basal invasion and distant spread of drosophila’s posterior intestinal cells (Bangi et al., 2013).
Gastric cancer	*H. pylori*, Lactic acid bacteria	The N-terminus of CagA could interreact with the tumor-suppressing protein and apoptosis-stimulating protein of p53 to subsequently disrupt the apoptotic function of the p53 tumor suppressor gene, which meant the possibility of progression to cancer was enhanced (Junaid et al., 2019); reduce the expression of the AU-rich element RNA-binding factor 1 *via* the CagA/p-ERK/AUF1 pathway to promote the incidence of gastric cancer (Guo Y. et al., 2020); the nucleotide transport and metabolism, amino acid transport and metabolism and the inorganic ion transport and metabolism were significantly abundant in the tumoral microbes (Liu et al., 2018); *H. pylori* penetrates the mucosal layer and settles on the surface of the gastric epithelial cells; releases toxic factors that damage the gastric epithelial cells; various inflammatory cells and mediators appear; produce immunoreactive substances, in addition to others (Goodwin, 1988). Lactic acid bacteria supply the exogenous lactate, which is a fuel source for cancer cells, promoting inﬂammation, angiogenesis, metastasis, epithelial-mesenchymal transition, immune evasion, production of reactive oxygen species and N-nitroso compounds (Vinasco et al., 2019).
HCC	*H. pylori*	The intrahepatic immune status and hemodynamics might be changed (Ki et al., 2010; Garcia et al., 2013; Okushin et al., 2018).
Cervical cancer	HPV, *Fusobacterium* spp.*, Chlamydia trachomatis, Atopobium vaginae, Dialister invisus, Finegoldia magna, Gardnerella vaginalis, Prevotella buccalis*, *P. timonensis*	Modify the tumor-immune microenvironment through the E-cadherin/β-catenin signaling pathway to cause cancer (Audirac-Chalifour et al., 2016); induce Th2 immunity through the RORγτ+Treg cells, IL-10, and Th17 cells in the cervical epithelium (Punt et al., 2015).
Ovarian cancer	*Chlamydia, Gemmata obscuriglobus*, *Halobacteroides halobius, Methyloprofundus sedimenti*, *Pediococcus*, *Pneumocystis*, *Acremonium*, *Cladophialophora*, *Malassezia*, Microsporidia *Pleistophora*, *Brucella*, *Mycoplasma*	Inhibiting apoptosis, inducing the DNA damage response and increasing the susceptibility to other infections (Shanmughapriya et al., 2012); disrupt genetic stability (Kidane et al., 2014); gene members of the homologous recombination repair pathway might have frequent genetic and epigenetic alterations in ovarian cancer; the deficiency in homologous recombination repair was shown to induce genomic instability and a hyper-dependence on alternative DNA repair mechanisms and to enhance the sensitivity of double-strand break-inducing agents (Konstantinopoulos and Matulonis, 2018); the metabolic characteristics have been found to be changed in the tumors, including the enriched and reduced metabolism (Li, 2020).
Head and neck squamous cell carcinomas	*Actinomycetes*, *Parvimonas*, Tissierellaceae	Without the secreting protease inhibitors that inhibited tumorigenesis (Hozumi et al., 1972).
OSCC	*Actinomyces* (Actinobacteria), Firmicutes (*Schwartzia* and *Selenomonas*), Spirochaetes (*Treponema*), *Porphyromonas gingivalis*	Promote the overexpression of nuclear factor kappa-light-chain-enhancer of activated B cells and the activation of cyclin-D1, an epidermal growth factor receptor ligand that promote the growth of tumors, eventually provoking nuclear translocation; epithelial mesenchymal transformation in malignant cells, tumor proliferation and tumor invasion (Hoppe et al., 2016; Lafuente Ibanez de Mendoza et al., 2019).

The number of microbes living on and inside the human body is about the same as the human cells (3.8×10^13^ bacterial cells relative to 3.0×10^13^ human cells) (Ron et al., 2016). Different microbes colonize different habitats in the human body, which can not only affect human health by aiding in nutrition, combating pathogens, modulating the immune system, and so on, but also be related to the high-risk of tumorigenesis (Dethlefsen et al., 2007; Cong and Zhang, 2018; Whisner and Aktipis, 2019). Because of the high mortality associated with malignant tumors (Whitlock et al., 2015; Collaborators, U.S.B.o.D et al., 2018; D’Souza et al., 2019), a new direction in therapy for treating microbes is innovatively put forward. Although possible relationships have been stated in various separate systems and pathogenic pathways, there is little correlative literature that sums up different cancers in a macroscopic view. Systematic information on microbes in different cancers has been hard to find.

This review aims to macroscopically summarize and verify the relationships between microbes (mainly bacteria) and tumoral *in situ* tissues from urinary, respiratory, digestive, reproductive, and other systems and to discuss the four major microbial pathogenic mechanisms related to carcinogenesis (the modulation of inflammation, immunity, DNA damage, and metabolism), as well as some synergistic pathogenic mechanisms (**Table 2**). We hypothesized that the regular monitoring of microbial changes might play a supervisory and manageable role in the development of cancer. This would represent a new challenge and opportunity for cancer prevention and treatment.

**Table 2:** The main mentioned cancers and microbes in this review from different pathogenic mechanisms.

Pathogenic mechanisms	Cancers	Microbes
Inflammation	Prostate cancer	–
	Lung cancer	*M. tuberculosis*
	Colorectal cancer	*Fusobacterium*
Immunity	Lung cancer	*M. tuberculosis*
	PDA	*-*
	Colorectal cancer	*Bacteroides*, *Fusobacterium*
	HCC	*H. pylori*
	Cervical cancer	*Fusobacterium* spp., HPV
Gene	CRC	*Salmonella enterica*
	Ovarian cancer	*Chlamydia*
	HCC	*H. pylori*
Metabolism	Lung cancer	*Thermus*, *Legionella*
	Gastric cancer	*H. pylori*
	HCC	*H. pylori*
	Ovarian cancer	*-*
	OSCC	*Porphyromonas gingivalis*
Synergistic pathogenesis	Prostate cancer	*Mycoplasma genitalium*
	Lung cancer	*M. tuberculosis*
	CRC	–
	HCC	–
	gastric cancer	*H. pylori*, lactic acid bacteria
	pancreatic cancer	–

## Relations Between Microbes in the In Situ Tissue and Tumors

### Prostate Cancer

A chronic persistent bacterial infection has been hypothesized as a possible etiology of prostate cancer due to the signs of inflammation in most precancerous and cancerous tissues (Lax and Thomas, 2002; Z, 2002; Nelson et al., 2003; Feng et al., 2019). To discover the relationship between microbes and prostate cancer, Feng et al. used integrated metagenomics and metatranscriptomics approaches to analyze 65 Chinese radical prostatectomy tumoral specimens and the adjacent benign tissues. The results showed that there was no microbial difference between the two groups in terms of alpha-diversity. This phenomenon was different from the expected difference, which might occur due to the close proximity of the compared regions and to the field effect. It was unclear whether the difference in genera or species existed (Feng et al., 2019).

However, Cavarretta et al. used extensive ultradeep pyrosequencing to detect the microbes in different tissues, including tumor, peri-tumor (PT), and non-tumor (NT) tissues from 16 white Caucasian non-diabetic nonobese prostate cancer patients who had not reported a prior known sexually transmitted infection or a recent history of urinary tract infection. Differences in terms of the microbial environment existed among the different zones of the prostate, especially the transition zone versus the peripheral zone. The microbes belonging to the PT region were more similar to the tumor region with respect to the NT region. This opposite result might be related to the rigorous entry standard for patients without other infectious diseases (Cavarretta et al., 2017).

To reduce the impact of close proximity of the compared regions and the field effect thoroughly, Miyake et al. examined 45 prostate cancer patients who underwent robotic prostatectomy and 40 benign prostatic hyperplasia patients. The rate of *Mycoplasma genitalium* infection in the prostate cancer patients was higher than in the benign prostatic hyperplasia patients (Miyake et al., 2019). The above microbial differences between the tumoral lesions and the nonneoplastic tissues indicated that microbes might be novel biomarkers and/or therapeutic targets for prostate cancer. In addition to being affected by microbes, the extracellular environment in prostate cancer might also conversely promote the establishment of specific microbes. The dysbiosis of the urinary microbes has not been fully confirmed as a cause or an effect for cancer (Cavarretta et al., 2017).

### Lung Cancer

The field of lung microbial studies is rapidly growing and has achieved provocative results regarding the relationship between lung microbes and cancer (Dickson et al., 2013; Goto, 2020; Sommariva et al., 2020; Xu et al., 2020). Because lung biopsy extraction from healthy people is unethical, some experiment data has been based on bronchoalveolar lavage, bronchoscopic brushing, or sputum samples, which have showed an abnormal increase in microbes in lung cancer (Hosgood et al., 2014; Perez-Losada et al., 2017; Tappenden et al., 2017). Laroumagne et al. showed that gram-negative bacteria, such as *Haemophilus inﬂuenzae*, *Enterobacter* spp. and *Escherichia coli*, colonized 39.8% of 216 lung cancer bronchoscopic samples (Laroumagne et al., 2011). Hosgood et al. discovered that *Granulicatella*, *Abiotrophia*, and *Streptococcus* were enriched in non-smoker lung cancer cases compared with healthy controls (Hosgood et al., 2014). Yan et al. compared the salivary samples of 20 lung cancer patients and 10 control subjects and discovered that *Capnocytophaga, Selenomonas, and Veillonella* were more abundant while *Neisseria* was less abundant in both squamous cell carcinoma and adenocarcinoma patients than controls. In addition, the combination of *Capnocytophaga* and *Veillonella*, two bacterial biomarkers, performed well in the prediction of squamous cell carcinoma and adenocarcinoma, which might be related to lung cancer screening (Yan et al., 2015). In a study with 10 sputum samples, *Streptococcus viridans* was more abundant in lung cancer patients than controls. Other sixteen bacterial species were found only in lung cancer patients, meanwhile 7 other species were revealed only in controls (Cameron et al., 2017). Lee et al. discovered that two phyla (*Firmicutes* and *TM7*) and two genera (*Veillonella* and *Megasphaera*) were relatively abundant in broncho alveolar lavage fluid of lung cancer patients (Lee S. H. et al., 2016).

However, samples from these alternative locations might contain contamination from the upper respiratory tract. The analysis of lung tissues has provided a more accurate assessment of microbes in lung cancer (Tappenden et al., 2017; Mao et al., 2018). H.-X. Liu et al. chose 24 patients with lung cancer, collecting cancerous sites and contralateral noncancerous sites as paired samples, and 18 healthy controls undergoing bronchoscopies. The microbial alpha diversity declined steadily from the healthy sites to the noncancerous sites to the cancerous sites. At the genus level, *Streptococcus* was more abundant in the cancer samples than in the controls, while *Staphylococcus* was more abundant in the controls (Liu H. X. et al., 2018). Yu et al. discovered that the alpha diversity in lung tumor tissues was lower than normal tissues, which was the same as the discovery by Jin J et al. (Yu et al., 2016; Jin et al., 2019). The phylum Proteobacteria (predominantly genera *Acinetobacter* and *Acidovorax*) showed a higher abundance and phylum Firmicutes (genus *Streptococcus*) and Bacteroides (genus *Prevotella*) exhibited a lower abundance in patients with lung cancer than emphysema (Liu Y. et al., 2018). In addition to nonmalignant and tumoral tissues, different types of lung cancer also showed a big difference in microbial content. Adenocarcinoma tumor tissues were found to have a significantly higher phylogenetic diversity, an increased relative abundance of *Thermus* and a decreased relative abundance of *Ralstonia* in comparison to squamous cell carcinoma (Yu et al., 2016). *Acinetobacter, Propionibacterium, Phenylobacterium, Brevundimonas*, and *Staphylococcus* were the major genera of adenocarcinoma, while *Enterobacter, Serratia, Kluyvera, Morganella, Achromobacter, Capnocytophaga*, and *Klebsiella* in squamous cell carcinoma. *Legionella* was found to be higher in patients with metastatic tumors (Yu et al., 2016; Gomes et al., 2019). Greathouse et al. detected that *Acidovorax, Klebsiella, Rhodoferax*, and *Anaerococcus* bacteria showed a significantly higher abundance in squamous cell carcinoma than in adenocarcinoma (Greathouse et al., 2018). The abovementioned differences between tumoral and noncancerous tissues and between different types of lung cancer, whether in samples of bronchoalveolar lavage, bronchoscopic brushing, sputum or lung tissues, have greatly advanced microbial studies of the development of lung cancer (Hosgood et al., 2014; Perez-Losada et al., 2017; Tappenden et al., 2017; Mao et al., 2018).

### Pancreatic Cancer

To learn more about pancreatic cancer, some studies have pointed out the possible existing relationship between microbes and tumors (Siegel et al., 2018). The specific microbial identification might be helpful for the early detection and treatment of pancreatic cancer (Zhang et al., 2019b).

Ertz-Archambault et al. concluded that *Helicobacter pylori (H. pylori)*, only for cag-A negative *H. pylori* strains but not cag-A positive, and *Fusobacterium* were possibly the major microbes affecting pancreatic cancer incidence (Ertz-Archambault et al., 2017). Mitsuhashi et al. observed 283 formalin-fixed, paraffin-embedded tissue specimens of pancreatic cancers. *Fusobacterium* was detected in 8.8% of the tumor tissues. The *Fusobacterium* species in the tumor was associated with a shorter survival in patients with pancreatic cancer (Mitsuhashi et al., 2015). However, Fan et al. supposed that *Fusobacterium* and *Lepotrichia* were protective and associated with a reduced risk of pancreatic cancer (Fan et al., 2018). The diverse results should also be drawn attention (Mitsuhashi et al., 2015; Fan et al., 2018). Nilsson et al. detected the DNA of enteric strains of *Helicobacter* that colonized the pancreas in 75% of the adenocarcinoma patients, 57% of the patients with neuroendocrine cancer, 38% of the patients with multiple endocrine neoplasia, 60% of the patients with chronic pancreatitis, but not in the control patients with benign disease or normal pancreas (Nilsson et al., 2006; Ertz-Archambault et al., 2017). Smruti Pushalkar et al. observed a greater presence and a larger abundance of bacteria in both mouse and human pancreatic ductal adenocarcinoma (PDA) tissues compared with normal pancreas tissues (Pushalkar et al., 2018). Berk Aykut et al. discovered that there were differences in the composition of fungi in tumoral samples and normal pancreatic samples. The *Malassezia* species was enriched in PDA tissues in both mice and humans. In addition, ablation of the microbes could prevent the progression of PDA and repopulation of the *Malassezia* species. The presence of *H. pylori* and *Malassezia* in pancreatic cancer was possibly due to passage of the intestinal flora through the pancreatic duct (Matsuda et al., 2009; Aykut et al., 2019).

### Gastric Cancer

*H. pylori* is a type of diverse bacterium with several virulent strain variations. The presence of *H. pylori* is mainly responsible for gastric inflammation and ulceration and signiﬁcantly increases the risk of gastric cancer. This phenomenon was discovered by Barry J. Marshall and J. Robin Warren, who were awarded the Nobel Prize in physiology for this discovery (Tan and Wong, 2011; Kalaf et al., 2013; Chen et al., 2014).

Uemura et al. chose 1,526 Japanese patients with duodenal ulcers, gastric ulcers, gastric hyperplasia, or non-ulcer dyspepsia who underwent endoscopy with biopsy at enrollment and then again at between 1 and 3 years after enrollment. After the follow-up period of 7 years, 36 gastric cancers (2.9%) were demonstrated in 1,246 patients with *H. pylori* infection and none in 280 patients without *H. pylori* infection. It appears that gastric cancer rarely occurs in the absence of *H. pylori* infection. Continuous *H. pylori* infection has been shown to lead to a high risk of gastric cancer (Luan et al., 2019). With the widespread recognition that *H. pylori* leads to gastric cancer, people have paid more attention to the eradication of *H. pylori*, and the use of quadruple drug therapy is common (Kim, 2019). Lee et al. conducted a meta-analysis to discover the effectiveness of treating *H. pylori* infection. Individuals who underwent an eradication of *H. pylori* demonstrated a lower incidence of gastric cancer than those who did not receive eradicative therapy. This result helped to confirm the therapeutic effectiveness of eradicating *H. pylori* (Lee Y. C. et al., 2016).

### Colorectal Cancer

Because the nonuniform bacteria present in different regions and stages of colorectal cancer, bacteria might play a role in prediction of early versus invasive cancer (Zorron Cheng Tao Pu et al., 2019). In 1971, Moore et al. analyzed the human fecal floras to know whether bacterial species were directly associated with high risk of colon cancer. However, this project was discontinued due to the complexity of the human intestinal flora and the limitations of statistical and analytical methods (Finegold et al., 1974; Moore and Holdeman, 1974; Holdeman et al., 1976). Since then, microbes had been investigated and recognized as major environmental factors relevant to colorectal cancer all over the world. Moore et al. reported that 15 bacterial species were significantly associated with a high risk of colorectal cancer in 1995 (Moore and Moore, 1995). Iradj Sobhani et al. used pyrosequencing to detect the bacterial genes and reported on the bacterial differences between cancer tissues and normal tissues. *Bacteroides* and *Prevotella* revealed an elevated level in cancer compared to normal groups, while other microbes showed no obvious difference (Sobhani et al., 2011). Kostic et al. characterized the composition of the microbes in colorectal cancer using whole genome sequences from nine tumoral and normal pairs. *Fusobacterium* sequences were enriched in carcinomas, while the Bacteroidetes and Firmicutes phyla were depleted (Kostic et al., 2012). Castellarin et al. reported that the presence of *Fusobacterium nucleatum* DNA was 415 times greater in tumor tissues than adjacent normal tissues, which might be associated with colorectal cancer (Castellarin et al., 2012). However, in a replication study, *Fusobacterium nucleatum* was detected in only 25% of colorectal carcinomas, which had no significant difference between tumor and adjacent tissues. This conflicting results might due to the differences of the populations or the technical issues (Repass et al., 2016). The following replication study showed that the difference in *Fusobacterium nucleatum* expression between colorectal cancer and adjacent normal tissues was smaller than the original research, and this bacterium was not detected in most samples, which could not confirm the results previously published, either. It was waiting to be solved whether the method of qPCR and primer and probe sequences, similarities, and differences in patient characteristics (Klevorn and Teague, 2016), and influence of diet on the gut microbiota (Mehta et al., 2017) could affect the result of this study (Repass et al., 2018). Just as for tumoral and normal pairs, the early and invasive colorectal cancer had different composition of microbes. *Oribacterium*, *Bacterioides*, and *Prevotella* were enriched in early cancer. Meanwhile, *Bacterioides*, *Oribacterium*, and *Fusobacterium* were found more abundant in the invasive cancer samples. The relative abundance of *Fusobacterium* increased in invasive cancer samples compared with early cancer samples in this study (Kostic et al., 2012; Haruki et al., 2019). *Escherichia coli (E.coli)*and *Bacteroides fragilis* were frequent, persistent mucosal colonizers of the familial adenomatous polyposis (FAP) gastrointestinal tract (2018). *Citrobacter rodentium* infection has been shown to promote the development of colon tumors in a murine model (Tan and Wong, 2011). The Apc ^min/+^mice (the mice model with adenomatous polyposis coli (APC) gene mutation) gavaged with feces from colorectal cancer patients had more intestinal tumors compared with healthy controls or the mice gavaged with phosphate-buffered saline (Li et al., 2019).

### Cervical Cancer

The changeable factors altering the vaginal microenvironment, such as bacterial vaginosis (Guo et al., 2012) or sexually transmitted infections (Vriend et al., 2015), have been identified as cofactors in persistent human papillomavirus (HPV) infections (Clarke et al., 2012). The bacterial differences of diversity between cancerous and noncancerous tissues had been discovered. Tango et al. discovered that nine genera and 21 species were significantly more abundant in cervical cancer group compared to the controls (Tango et al., 2020). Audirac-Chalifour et al. selected 32 cases containing 20 non-cervical lesions (NCL: 10 HPV-negative; 10 HPV-positive), four squamous intraepithelial lesions (SILs: all HPV-positive), and eight cervical cancer lesions (CCL: all HPV-positive) to analyze the alpha-diversity and the β-diversity in each group. HPV-negative NCL showed a significant difference in the diversity of the bacteria with SIL and CCL. The CCL samples showed a higher diversity compared with the NCL-HPV negative samples. *Sneathia* spp.*, Megasphaera elsdenii*, and *Shuttleworhia satelles* were most representative in SIL. The most abundant species in the cervix of women with SIL was *Sneathia* spp., which was less abundant in patients with cervical cancer, whereas the most abundant species was *Fusobacterium* spp. in CCL samples and *L. crispatus* and *L. iners* in samples with a normal cytology. (Audirac-Chalifour et al., 2016). Not only cervical microbes but also abnormal vaginal ones were associated with the development of cervical cancer (Guijon et al., 1992). The *Chlamydia trachomatis* were reported to be a possible co-factor for cervical cancer development (Lehtinen et al., 2011). So et al. exhibited that the diversity of vaginal microbes was higher in cervical cancer than normal women. The proportion of *Lactobacillus* species were decreased in cervical disease. The abundance of *L. crispatus* in vagina was significantly reduced in cervical cancer. The infections of *Atopobium vaginae, Dialister invisus, Finegoldia magna, Gardnerella vaginalis, Prevotella buccalis*, and *P. timonensis* were significantly associated with the high risk of high-grade squamous intraepithelial neoplasia and cervical cancer (So et al., 2020). The depletion of *Lactobacillus* spp. and overgrowth of diverse obligate and strict anaerobic bacteria were supposed to be relevant to an increased risk of HPV acquisition and decreased clearance of HPV (Watts et al., 2005; Gillet et al., 2011; Guo et al., 2012).

### Ovarian Cancer

The miscellaneous different microbes in ovarian cancers and control samples indicate that they might affect the microenvironment of ovarian tissues. Shanmughapriya et al. discovered that *Chlamydia* were present in 70% of the ovarian cancer tissues compared with healthy controls (Shanmughapriya et al., 2012). Li et al. proposed that the composition of bacteria and archaea was significantly different between cancerous and noncancerous ovarian tissues in situ. Aquificae and Planctomycetes were found to be increased in cancerous tissues, while the Crenarchaeota were found to decreased when compared to noncancerous tissues at the phylum level. In cancerous tissues at the class level, Spartobacteria were found to be increased and Sphingobacteriia were found to be reduced. The phylum, class, order, family, genus and species level of bacteria were all found to be significantly different between cancerous and noncancerous tissues. At the species level, *Gemmata obscuriglobus* dominated the cancer group, followed by *Halobacteroides halobius* and *Methyloprofundus sedimenti*. *Halobacteroides halobius* dominated the control group, followed by *Gemmata obscuriglobus* and *Methyloprofundus sedimenti*. In genus level, the relative abundance of *Paenibacillus, Haloferula, Subdivision, Zavarzinella, Photorhabdus, Volucribacter, Blastococcus, Mesotoga, Defluviitoga*, and *Dorea* was significantly different between cancer and control groups. Particularly, the relative abundance of *Anoxynatronum sibiricum* might be associated with the stage of tumor (Li, 2020). Banerjee et al. used PathoChip, a very sensitive approach to detect human pathogenic microorganisms, to screen the microbes in 99 ovarian cancer samples and 20 matched (tissues adjacent to the tumor deemed to be noncancerous), as well as in 20 unmatched control samples. Proteobacteria and Firmicutes were found in the majority of all three samples, as well as Bacteroidetes, Actinobacteria, Chlamydiae, Fusobacteria, *Spirochaetes*, and Tenericutes at lower percentages. The composition of microbes were quite different among the ovarian cancer samples, matched samples and unmatched samples. The bacterial signatures detected in the cancer samples were significantly higher than the controls. *Pediococcus* signatures were detected with the highest hybridization signal in the ovarian cancer samples, closely followed by *Burkholderia*, *Sphingomonas*, *Chryseobacterium*, *Enterococcus*, *Staphylococcus*, *Treponema*, *Francisella*, and *Shewanella* signatures. For other microbes, signatures of *Pneumocystis*, *Acremonium*, *Cladophialophora*, *Malassezia*, and Microsporidia *Pleistophora* were detected in all ovarian cancer samples. *Rhizomucor*, *Rhodotorula*, *Alternaria*, and *Geotrichum* were shown in more than 95% of ovarian cancer samples. *Brucella*, *Chlamydia* and *Mycoplasma* were detected in 76%, 60%, and 74% of the ovarian cancer samples, respectively. The viral sequences were integrated widely into the genome of the tumor tissue, especially the high risk HPV16 and 18 along with other low risk HPVs. The integration of HPV genomic regions into the human genome had been considered as an important event in cancer development (Funda, 2007; Al-Shabanah et al., 2013; Kadhim et al., 2014). Merkel cell Polyomaviruses were significantly detected in the ovarian cancers but undetectable in the controls (Banerjee et al., 2017). Herpesviruses (Shanmughapriya et al., 2012; Pandya et al., 2014), and Retroviruses (McLaughlin-Drubin and Munger, 2008; Johal et al., 2010) had been detected in 50% and 16%, respectively, of ovarian cancers. The specific retroviral probes in the majority of cancers were Mammary Tumor Virus and Foamy Virus. The genomic integration of human herpesviruses -6a at the telomeric region could be a contributing factor to ovarian cancer (Banerjee et al., 2017). The abnormal microenvironment might influence the incidence and progression of ovarian cancer. However, it cannot be denied that the specific tumoral environment might also provide a specialized niche for the microbial colonization (Banerjee et al., 2017).

### Other Cancers

In squamous cell carcinomas, such as head and neck squamous cell carcinomas and oropharyngeal squamous cell carcinoma (OSCC), a difference of the relative abundance and diversity in the microbes between tumoral and normal tissues is proposed to exist, which reveals the possible relationships between microbes and cancers in most parts of the human body (Hozumi et al., 1972; Wang et al., 2017; Wolf et al., 2017; Zhao et al., 2017). Wang et al. compared the relative abundance of individual taxa between tumoral and normal tissues in head and neck squamous cell carcinomas. For not only *Actinomycetes* themselves, but also the parent taxa of *Actinomycete* which could be up to the phylum level, their relative abundance was significantly relatively increased in low-stage patients compared with high-stage patients. *Actinomycetes* might play a protective role by secreting protease inhibitors that inhibit tumorigenesis (Hozumi et al., 1972). In contrast, the *Parvimonas* genus and its family Tissierellaceae were found to be increased in tumors relative to normal tissues. These differences were more pronounced in patients with a higher T stage (Wang et al., 2017).

Of the human oral microbes, there are probably 500 to 700 common oral species or phylotypes, which might impact the luminal system to lead to OSCC (Samaranayake and Matsubara, 2017; Wolf et al., 2017). The oral microbes showed a significant difference between cancer patients and healthy humans. Wolf et al. analyzed saliva samples from 11 patients with OSCC and 11 healthy controls by high-throughput sequencing of the 16S rRNA gene using the MiSeq platform. Signatures of Bacteroidetes (e.g. *Prevotella*), Proteobacteria (e.g. *Haemophilus* and *Neisseria*), and Firmicutes (e.g. *Streptococcus* and *Veillonella* ) were more abundant in the healthy controls, whereas signatures of Actinobacteria (e.g. *Actinomyces*), Firmicutes (e.g. *Schwartzia* and *Selenomonas*), and Spirochaetes (e.g. *Treponema*) were more abundant in the OSCC patients (Wolf et al., 2017). Zhao et al. observed 40 tumoral and matched normal control samples from the same individual with OSCC. Compared with the corresponding clinically normal control samples, the diversity of the bacterial community in the cancer samples was significantly increased. The lesion surface of OSCC showed a higher phylogenetic diversity. The bacterial taxa between the cancer and control samples showed the opposite relationships. This implies that the enormous changes of bacterial symbiotic relationships might be associated with the occurrence of OSCC (Zhao et al., 2017).

## Different Mechanisms of Carcinogenesis

### Inflammation

Inflammation can result in a series of pathological changes, such as cancer and fibrosis (Hanahan and Weinberg, 2000; Karin and Clevers, 2016). Soluble and cellular inflammatory mediators are responsible for tumor development and progression (Galdiero et al., 2017). Up to 10%–20% of all cancers could be attributed to infections (Grivennikov et al., 2010). The following results showed some cancers that were related to inflammation caused by microbes.

In recent studies, human microbes might influence the initiation and/or progression of prostate cancer through both direct and indirect interactions, such as infecting and inducing both symptomatic and asymptomatic inflammatory responses in the prostate (Yoon et al., 2012; Caini et al., 2014; Cavarretta et al., 2017).

The distribution of microbes in the lung has been shown to be affected by many factors, such as environment and geography (Ubags and Marsland, 2017). Pre-existing *Mycobacterium tuberculosis* might increase the risk of lung cancer (Ardies, 2003; Khoruts, 2018). The relationship between *M. tuberculosis* and lung cancer was proposed based on previous studies of chronic inﬂammation-associated carcinogenesis, showing increase in tumor necrosis factor and excessive and persistent local inflammation at sites of repair and fibrosis (Coussens and Werb, 2002; Ardies, 2003). Some cytokines might promote the survival of malignant cells as autocrine growth factors (Mantovani, 2001).

The animal and human studies showed that inflammation might have an impact on the development of colorectal cancer (de Visser et al., 2006; Kuraishy et al., 2011; Kux and Pitsouli, 2014; Karin and Clevers, 2016; Li et al., 2019). Microbes in colorectal cancer patients might destroy the intestinal barrier, leading to the subsequent induction of pro-inﬂammatory cytokines, such as the reactive oxygen species (ROS), which promoted regeneration and predisposed to tumorigenesis (Kuraishy et al., 2011; Kux and Pitsouli, 2014; Li et al., 2019). The blood cell infiltration was thought to be induced by intestinal inflammation and tumor-promoting inflammation in mammals possibly (de Visser et al., 2006). It was reported that the tissue regeneration caused by inflammation might play a role in inflammation-driven carcinogenesis. Inflammation due to microbial pathogens could interfere with signaling pathways to affect several cytokines and growth factors, such as IL-6, IL-2, and tumor necrosis factor, to control the process of regeneration in injured intestinal mucosa. The signaling pathways included mitogen-activated protein kinase–AP-1, IKK–NF-κB, Hippo–YAP, Notch, the Epidermal Growth Factor Receptor, Target-of-rapamycin/Vascular Endothelial Growth Factor Receptor, JAK/STAT, and Wnt/Wg signaling, and so on (Stavria and Yiorgos, 2013; Kux and Pitsouli, 2014). Inflammation and cancer might affect and promote each other. The cancer cells could also express inflammatory cytokines in turn to recruit immune cells to cause tumor-related inflammation (Kuraishy et al., 2011). *Fusobacterium* sequences were enriched in carcinomas (Kostic et al., 2012), which could bind to host epithelial Cadherin 1 through the adhesion of FadA and invade epithelial cells from through the E-cadherin/β-catenin signaling to induce inflammation and tumor cell growth in transformed cells (Rubinstein et al., 2013; Wong and Yu, 2019; Guo P. et al., 2020).

### Immunity

Microbes might affect the immune status to promote the development and progression of several cancers, as described in a number of studies (Rooks and Garrett, 2016; Goodman and Gardner, 2018; Pushalkar et al., 2018). Different immune status can promote the tumor progression. Cancer and cancer-related disease can be affected by the immune status in turn. For example, the occurrence of tuberculosis in lung cancer patients might be accelerated after chemotherapy due to low immunity (Christopoulos et al., 2014; Khoruts, 2018; Pandey et al., 2019). A low immune status that means immunosuppression is one of the potential mechanisms of microbial carcinogenesis that allow the tumor to grow and infiltrate (Shalapour and Karin, 2019). The tumor-associated macrophages play a key role in inhibiting T cell recruitment and function, thus facilitating tumor immune escape (Quaranta and Schmid, 2019). Not only the low but also the wrong and excessive immune status could affect the incidence of cancer. Once the innate immunity is activated, the MHC class I and II and costimulatory molecules are upregulated, as well as numerous inflammatory chemokines and cytokines, then the adaptive immune cells are activated to amplify the initial inflammatory response (Monney et al., 2002; Steinman, 2012). Tumor-associated neutrophils could release ROS and neutrophil elastase to mediate cancer cell killing and promote metastasis (Galdiero et al., 2013). Tumor-associated macrophages could induce the wrong immune reaction to play a supportive role in cancer development and progression (Galdiero et al., 2013; Ruffell et al., 2014)

In PDA, it was demonstrated that microbes might activate selected toll-like receptors (TLR) in monocytic cells to generate a tolerogenic immune program. TLR2 and TLR5 ligation was demonstrated to induce innate and adaptive immune suppression, which promote PDA. Without the TLR signaling, the immune suppression by the macrophages would not occur. Therefore, the management of microbes to reduce immune suppression might be an attractive method for pancreatic cancer treatment (Pushalkar et al., 2018).

The importance of gut microbes in intestinal carcinogenesis in modulating the tumor immune microenvironment has been improved by several researches (Rooks and Garrett, 2016; Goodman and Gardner, 2018; Gopalakrishnan et al., 2018; Routy et al., 2018; Tilg et al., 2018). In MSI-high colorectal cancer, the abundant neoantigens due to a large number of transcoding mutations could lead to a strong immune response to the tumor (Ogino et al., 2009; Nosho et al., 2010; Rizvi et al., 2015; Chantziantoniou et al., 2017; McDermott et al., 2017). The microbes in ileum could transform the tolerogenic apoptosis of ileal intestinal epithelial cells into immunogenic cell demise, then elicit IL-1β-dependent follicular T helper responses (Roberti et al., 2020). As for the sustained survival of colorectal carcinoma (CRC) cells, *Fusobacterium* could exert an immunosuppressive effect in the cancer microenvironment (Saito et al., 2016; Thiele Orberg et al., 2016; Hussan et al., 2017; Park et al., 2017; Ye et al., 2017). IL17 immune cells and *Bacteroides* were found to have infiltrated the majority of the tumor samples and the lamina propria of homologous normal mucosa in cancer patients, while they were rarely or not detected in the mucosa of normal individuals. It was concluded that *Bacteroides* might change the immune microenvironment relative to the incidence of cancer (Sobhani et al., 2011). Jasemi et al. classified *Bacteroides fragilis* into three groups based on biofilm formation ability and toxin encoding gene presence and its flanking region. Enterotoxigenic *Bacteroides fragilis* strains with *Bacteroides fragilis* toxin gene was deﬁned as pattern I, and non-toxigenic strains were deﬁned as Pattern II and III. Pattern II was deﬁned as strains without the pathogenicity island region and ﬂanking region. Pattern III was deﬁned as strains without the pathogenicity island and with ﬂanking region. The abundance of patterns were different between CRC and normal tissues, which meant pattern I > III > II in normal tissue and pattern II > III > I in CRC tissue. The strains isolated from CRC tissue had higher biofilm forming ability than the strains isolated from normal tissue (Jasemi et al., 2020). Intestinal mucosal IL17A could not only protect the intestine from pathogens but also provide transition to promote CRC development. Chronic mucosal formation of IL17A produced by Th 17 cells might alter signaling pathways in colon epithelial cells or induce changes or mutations in DNA structure that facilitated the transformation of colon epithelial cells contributing to carcinogenesis (Hurtado et al., 2018).

*H. pylori* has been shown to play a possible role in hepatocellular carcinoma (HCC) because the *H. pylori* genes frequently has been detected in resected HCC specimens (Guo et al., 2012; Mitsuhashi et al., 2015; Lee Y. C. et al., 2016; Okushin et al., 2018; Haruki et al., 2019). Some *in vitro* studies using cancer cell lines discovered an indirect pathogenesis, possibly including immune changes, due to *H. pylori* in HCC (Zhang et al., 2005; Ito et al., 2008; Pekkala et al., 2019). HCC is usually accompanied by liver fibrosis in which the intrahepatic immune status and hemodynamics might be changed, leading to the inflow of *H. pylori* and escape from immunity in the liver. However, it was not confirmed whether *H. pylori* played a causative role in the development of HCC. In animal experiment, *H. pylori* infection did not promote the development of HCC in transgenic mice expressing HCV proteins (Ki et al., 2010; Garcia et al., 2013; Okushin et al., 2018). Therefore, a correlation between *H. pylori* infection and HCC was not strongly supported. Further experiment should be performed.

Microbes might produce immune substances to modulate the development of SIL and cervical cancer, which could be potential biomarkers in cervical cancer (Audirac-Chalifour et al., 2016). Specific bacteria might induce Th2 immunity through the RORγτ+Treg cells, IL-10, and Th17 cells in the cervical epithelium (Punt et al., 2015). The activities of these substances induced an abnormal state in the epithelial cells which promoted the incidence of cervical cancer (Bermudez-Morales et al., 2008; Punt et al., 2015).

Cancer immunotherapy has become increasingly sophisticated, especially cancer vaccines that stimulate the active immune system to target the tumor neo-antigens, which are unique to each patient’s tumor, and to facilitate recognition and elimination of transformed cells by the immune system (Fioretti et al., 2014; Mannan, 2016; Hobernik and Bros, 2018; Pandey et al., 2019; Shukla et al., 2020). For example, the creation of the HPV vaccine is well known for the prevention of cervical cancer. For the vaccines using recombinant DNA technology to prevent HPV infection, there are three types at present, including a bivalent vaccine against HPV16 and HPV18 (Cervarix), a tetravalent vaccine against HPV6, 11, 16, and 18 (Gardasil), and a nonavalent vaccine against HPV6, 11, 16, 18, 31, 33, 45, 52, and 58 (Gardasil 9) (Garbuglia et al., 2020). The immunotherapy of breast cancer has been explored, including the use of targeted antibodies such as trastuzumab and pertuzumab. However, these antibodies are effective approaches only for patients whose tumors overexpress the human epidermal growth factor receptor 2 (HER2) antigen. For those patients without the HER2 antigen, these antibodies might not be useful (Coussens and Werb, 2002; Combadiere and Mahe, 2008; Mittendorf et al., 2011; Berger and Wunderink, 2013; Mattheolabakis et al., 2016). Therefore, a vaccine for breast cancer has been explored as an alternative to support the unmet needs of such patients, such as a particulate vaccine delivered *via* the skin, which was found to be a better route of immunization in a murine model (Weitzman and Gordon, 1990).The vaccines mentioned above were shown to be effective in cancer immunotherapy. Therefore, new cancer vaccines would greatly progress the treatment of cancer (Mattheolabakis et al.; Mittendorf et al.; Coussens and Werb, 2002; Combadiere and Mahe, 2008; Berger and Wunderink, 2013; Chablani et al., 2019; Garbuglia et al., 2020).

### DNA Damage

Genetic changes, mainly those associated with DNA damage and repair, are known to be linked to cancer. If the DNA damage induced by microbes is not appropriately repaired, this might lead to mutations and genomic instability, eventually leading to cancer (De Bont and van Larebeke, 2004; Trabulus et al., 2012; Kidane et al., 2014). All of the following cancers involved DNA damage including CRC, ovarian cancer, HCC, gastric cancer, and lung cancer (Nalbandian et al., 2009; Shanmughapriya et al., 2012; Konstantinopoulos and Matulonis, 2018; Junaid et al., 2019b; Martin et al., 2019).

The enrichment of bacterial families in the CRC microbes is shown to produce genotoxins inducing damage in the host cell DNA, which might possibly lead to colorectal cancer. Martin et al. discovered that infection with *Salmonella enterica*, a genotoxin-producing bacterium, could enhance genomic instability both in two-dimensional and organotypic three-dimensional tissue models in FAP and the majority of sporadic CRC (Fearon, 2010). The APC gene might be lost after exposure to the genotoxic bacteria. The deficiency in APC is associated with the sustained activation of the DNA damage response and the reduced capacity to repair different types of damage, including DNA breaks and oxidative damages. Infection with genotoxic *Salmonella* was shown to prevent cell cycle arrest in APC-deficient cells (Martin et al., 2019). The cytolethal distending toxin produced by *Escherichia* and *Campylobacter* spp. could induce double-strand DNA break *via* its deoxyribonuclease activity to develop cancer (Cuevas-Ramos et al., 2010; He et al., 2019). Colibactin produced by members of the Enterobacteriaceae family could also induce DNA strand break (Buc et al., 2013). *B. fragilis* toxin (Goodwin et al., 2011) and ROS produced by *E. Faecalis* were both associated with DNA damage and genomic instability *in vitro* (Huycke, 2002; Wang and Huycke, 2007).

In ovarian cancer, *Chlamydia* were found to exist in tumor tissues, which might contribute to cancer through inhibiting apoptosis, inducing the DNA damage response and increasing the susceptibility to other infections (Shanmughapriya et al., 2012). Like *Chlamydia*, microbes might disrupt genetic stability to increase the incidence of ovarian cancer (Kidane et al., 2014). Large-scale genomic studies have shown that gene members of the homologous recombination repair pathway might have frequent genetic and epigenetic alterations in ovarian cancer. The deficiency in homologous recombination repair was shown to induce genomic instability and a hyper-dependence on alternative DNA repair mechanisms and to enhance the sensitivity of double-strand break-inducing agents (Konstantinopoulos and Matulonis, 2018).

As far as we know, *H. pylori* is related to HCC and gastric cancer. The cytotoxin-associated gene (Cag) pathogenicity island, which encodes a type IV secretion system that injects CagA into epithelial cells, is well known among the *H. pylori* virulence factors (Censini et al., 1996; Odenbreit, 2000; Asahi et al., 2000; Stein et al., 2000; Lai et al., 2006; Moore et al., 2011). The N-terminus of CagA could interreact with the tumor-suppressing protein and apoptosis-stimulating protein of p53 to subsequently disrupt the apoptotic function of the p53 tumor suppressor gene, which meant the possibility of progression to cancer was enhanced (Junaid et al., 2019).

Regional tumor peptides and even radiotherapy might lead to a microenvironment deregulation in granulomas, allowing the consequent proliferation of *M. tuberculosis* (Christopoulos et al., 2014). Chronic *M. tuberculosis* infection in a mouse model also induced squamous cells to aggregate in the lung, with malignancy and tumorigenicity possibly due to the DNA damage. The accumulation of DNA damage would be malignant and ultimately lead to lung cancer (Nalbandian et al., 2009).

### Metabolism

Tumor cells must increase the import of nutrients from their environment to maintain a high metabolic level, due to their rapid growth compared with normal or quiescent cells, by adapting their metabolism to be more dependent upon aerobic glycolysis and glutaminolysis (Kidane et al., 2014; Deberardinis and Chandel, 2016; Weyandt et al., 2017). Warburg effect is the switched metabolism existing in cancerous cells, which primarily undergo aerobic glycolysis instead of oxidative metabolism. The tumor cells express increased glucose uptake and elevated lactate production different from the normal metabolism. Butyrate is the primary energy source of normal colonocytes, which could accumulate and inhibit the proliferation of cancerous colonocytes when the Warburg effect happens. Cancer cell metabolism could drive the divergence of epigenomic and transcriptome profiles in cancer cells away from their original cells and promote tumor development (Racker, 1972; Vander et al., 2009; Donohoe et al., 2012). The metabolic reprogramming of tumor cells including altering bioenergetics, enhancing biosynthesis, and redoxing balance improve cell fitness and provide a selective advantage during tumorigenesis (Deberardinis and Chandel, 2016). These activities enhance or suppress in tumor cells compared with normal metabolism which has a stable anabolic program (Deberardinis and Chandel, 2016). The tumor cells have various mechanisms to maintain the viability in nutrient- and oxygen-poor environments, such as decreasing their demand for ATP or stimulating ATP production by activating adenylate kinase (Strohecker and White, 2014; Deberardinis and Chandel, 2016). For microorganisms, all of them must regulate the uptake of nutrients and coordinate the metabolism of carbon, energy, and nitrogen (Chubukov et al., 2014). The normal microbe-derived metabolites containing SCFAs (particularly acetate, propionate, and butyrate) have the robust capacity to dampen intestinal inflammation, protect against pathogen invasion and maintain barrier integrity. Once this process is disrupted, the abnormal metabolism might affect the tumor growth (Zhang et al., 2019a). The metabolic substances, which helps cells acquire the energy that they need for growth, proliferation, and the maintenance of critical cellular processes, can support the biological processes that enable tumor growth (Vander Heiden and DeBerardinis, 2017; Weyandt et al., 2017). In addition, many microbes can produce toxins, specifically those that disrupt cellular signaling, to disturb the regulation of cell growth (Wei et al., 2012). As the metabolic profiles of tumor cells distinguishes them from normal cells and are responsible for their growth and survival, the metabolism would be innovative targets for the management and prevention of cancers (Weyandt et al., 2017).

In lung cancer patients, Ke et al. discovered that the metabolic characteristics of microbes were different in diverse sampling sites. Microbes in the tumors exhibited metabolic behaviors; antimicrobial resistance, protein folding, sorting and degradation, glycan biosynthesis and metabolism, metabolism of cofactors and vitamins and nucleotide metabolism were found to be enriched only in cancer patients (Wang et al., 2019). For example, airway microbes were observed to upregulate the phosphoinositide 3-kinase pathway, involved in the incidence of lung cancer, to participate in regulating cell proliferation, survival, and differentiation (Mendoza et al., 2011; Tsay et al., 2018). *Thermus* and *Legionella* were speculated to play a potential role in tumor progression, partially through different metabolic-related functions, such as reduced signal transduction, increased excretory systems, amino acid metabolism, aldosterone-regulated sodium reabsorption, or amoebiasis pathways (Gomes et al., 2019).

*H. pylori* has been shown to signiﬁcantly increase the risk of gastric cancer (Tan and Wong, 2011; Kalaf et al., 2013; Chen et al., 2014). Guo et al. concluded *H. pylori* infection might reduce the expression of the AU-rich element RNA-binding factor 1 *via* the CagA/p-ERK/AUF1 pathway to promote the incidence of gastric cancer (Guo Y. et al., 2020). Xiaosun, et al. revealed the differences in metabolism among tumoral, peritumoral, and normal tissues. The nucleotide transport and metabolism, amino acid transport and metabolism and the inorganic ion transport and metabolism were significantly more abundant in the tumoral microbes, which were relevant to gastric cancer (Liu et al., 2018).

*H. pylori* might induce the incidence of HCC through not only the immune pathway but also metabolic-related way. Ki et al. postulated that *H. pylori* infection might promote the TGF-β1-dependent oncogenic pathway to disturb the balance between hepatocyte apoptosis and proliferation in a murine model of CCl4-induced fibrosis (Ki et al., 2010; Garcia et al., 2013; Okushin et al., 2018). After the intestinal barrier is damaged, the gut microbes might putatively flow into the portal vein to settle into the liver (Okushin et al., 2018). Subsequently, these microbes might cause HCC through multiple mechanisms, including the release of cancer-promoting and senescence-promoting metabolites, such as deoxycholic acid from the dysbiotic microbes, and an increased hepatic exposure to gut-derived microbiota-associated molecular patterns, such as lipopolysaccharide. The above conditions might promote hepatic inflammation, fibrosis, proliferation, and the activation of anti-apoptotic signals in turn (Yu and Schwabe, 2017). HCC might be prevented from manipulating the gut microbes by diet, lifestyle, antibiotics and probiotics, due to the abovementioned pathogenic mechanisms (Mima et al., 2017).

In ovarian cancer, it was found 46 different Kyoto Encyclopedia of Genes and Genomes (KEGG) pathways in the bacteria of ovaries between cancer and control groups. The changeable metabolic characteristics in the tumors included increased pathways related to streptomycin biosynthesis, carbon fixation in photosynthetic organisms, glycosphingolipid biosynthesis-globo series, cyanoamino acid metabolism, glycerophospholipid metabolism, butirosin and neomycin biosynthesis, other glycan degradation, and so on. As for the decreased pathways in cancer tissue, the bacteria showed the reduced alpha-linolenic acid metabolism, biosynthesis of unsaturated fatty acids, sulfur metabolism, biotin metabolism, protein kinase activity, biosynthesis of ubiquinone and other terpenoid-quinones, two-component system, folate biosynthesis, cell motility and secretion, citrate cycle, and ribosome biogenesis in eukaryotes. These enrichments and reductions of pathways might be the consequence of the microbial effect, which were involved in the development of cancer (Li, 2020).

Lafuente Ibáñez de Mendoza et al. concluded that *Porphyromonas gingivalis* might play an important role in OSCC. The overexpressed defensins in the OSCC samples, which contained human neutrophil peptide-2 and human α-defensin, might promote the overexpression of nuclear factor kappa-light-chain-enhancer of activated B cells and the activation of cyclin-D1, an epidermal growth factor receptor ligand that promoted the growth of tumors, eventually provoking nuclear translocation. The possible mechanisms include epithelial mesenchymal transformation in malignant cells, tumor proliferation, and tumor invasion. However, the accuracy of this finding is still waiting to be confirmed in *in vitro* studies and animal models (Hoppe et al., 2016; Lafuente Ibanez de Mendoza et al., 2019).

### Synergistic Pathogenesis

Although the pathogenic mechanisms of microbes in cancer can be divided into the four main areas of modulating inflammation, immunity, DNA damage, and metabolism, microbes can induce the incidence of cancer through multiple pathogenic mechanisms. In other words, the abovementioned pathogenic mechanisms may not work absolutely independent of each other (Francescone et al. 2011; Bouvard et al., 2009; Kidane et al., 2014; Weyandt et al., 2017). One type of cancer might occur due to two or more of these microbial mechanisms, as suggested in the following studies. The different pathogenic mechanisms might overlap in a single cancer.

The metabolic substances released by microbes can induce inflammatory, immune, genetic, and metabolic responses. It has been estimated that over 25% of human cancers are related to a chronic inflammatory status, which might be a consequence of microbial infection and immunologic abnormality (Bouvard et al., 2009). The stressed or dying cells due to microbial infection recruit different types of immune cells including macrophages, neutrophils, T-cells, and B-cells to promote inflammation further to activate various tumor-promoting inflammatory cytokines (Kuraishy et al., 2011). Chronic inflammation could produce an immunosuppressive microenvironment to support tumor development and inhibit anti-tumor immunity (Shalapour and Karin, 2019). In prostate cancer, *Mycoplasma hyorhinis* might induce tumorigenesis not only indirectly through a partial chronic inflammatory response, but also directly through bacterial protein products, such as p37, that exert oncogenic effects (Ketcham et al., 2005; Goodison et al., 2007). In lung cancer, microbes might lead to malignancy through microbial dysbiosis, genotoxicity and virulence effects, inﬂammation, immune responses, and metabolism (Weitzman and Gordon, 1990; Coussens and Werb, 2002; Ballaz and Mulshine, 2003; Roesler et al., 2012; Liu H. X. et al., 2018). After damaging the pulmonary epithelium, the cytokines, released by infiltrating lymphocytes and macrophages, might induce a cytokine cascade and a proliferation of the lung epithelial cells. Subsequently, the breaks in the chromosomal strands and the accumulation of DNA mutational changes might be eventually activated by ROS (Weitzman and Gordon, 1990; Ballaz and Mulshine, 2003).

The gut microbes, which are well known in the development of colorectal cancer, induce various physiological functions, including cell proliferation, angiogenesis and apoptosis (Dolara et al., 2002; Stappenbeck et al., 2002; Rakoff-Nahoum and Medzhitov, 2007; Cheesman et al., 2011). The intestinal microbes in some sub-groups of colorectal cancer patients contain more bacteria that could produce metabolites or genotoxins, such as cytolethal distending toxin and colibactin, to result in gastrointestinal tract inflammatory diseases or to affect the tumorigenesis of colorectal cancer. Some of them can be directly pro-carcinogenic or opportunistic microorganisms in the tumor-associated microenvironment. For example, The cytolethal distending toxin produced by *Escherichia* and *Campylobacter* spp. (Cuevas-Ramos et al., 2010; He et al., 2019), Colibactin produced by members of the Enterobacteriaceae family (Buc et al., 2013), *B. fragilis* toxin (Goodwin et al., 2011) and ROS produced by *E. Faecalis* could induce the DNA strand break which might be associated with tumorigenesis (Huycke, 2002; Wang and Huycke, 2007). *E. coli* containing the polyketide synthase (pks) genotoxic island induced the DNA damage *in vitro* and *in vivo* and developed CRC in azoxymethane-treated IL-10 deficient mice (2012). After the cocolonization with pks+ *E. coli* and *Bacteroides fragilis*, the DNA damage was obviously enhanced in the mice colon epithelial cells. The mucus degradation increased adhesion of pks + *E. coli* and induced DNA damage in colon epithelial cells. *Bacteroides fragilis* promoted the IL-17 induction with early augmentation by pks+ *E. coli* cocolonization (Dejea et. al., 2018).

The interaction between microbes and colon tissue might activate the pro-carcinogenic signaling pathways and result in molecular changes, ultimately leading to cancer (Wong and Yu, 2019; Zorron Cheng Tao Pu et al., 2019). Human CRC tumors expressed more chemokine (C-C motif) ligand 2/monocyte chemoattractant protein 1 (CCL2/MCP-1) than the healthy colon sites. Satu Pekkala et al. discovered that the exposure of CRC cells to bacterial ﬂagellin increased interleukin 6 (IL6) and *CCL2/MCP-1* mRNA expression and IL6 excretion. Flagellin was shown to decrease caspase-1 activity and the production of ROS to increase cytotoxicity in CRC cells. Conditioned media from ﬂagellin-treated CRC cells deteriorated the C2C12-myotubes and decreased their numbers. Increased ﬂagellated microbes might promote CRC survival by inducing inﬂammatory proteins, including MCP-1 and others (Pekkala et al., 2019). In animal studies, evidence has suggested that microbes can directly contribute to the development of CRC through interaction with the immune system, the production of cancer-associated metabolites and the release of genotoxic virulence factors (Arthur et al., 2012; Kostic et al., 2013; Zackular et al., 2013). The *Bacteroides fragilis* toxin might be a risk factor for developing CRC, which upregulated the spermine oxidase (SMOX) gene expression in human normal colon epithelial cells (Goodwin et al., 2011). The SMOX protein played an important role in the alteration of polyamine metabolism, which catalyzed the oxidation of spermine to spermidine and produced hydrogen peroxide and aldehydes to result in apoptosis, DNA damage, and consequently the development of CRC (Goodwin et al., 2011; Snezhkina et al., 2016). In a drosophila model of gut pathogenesis, the intestinal infection with *Pseudomonas aeruginosa* could activate the c-Jun N-terminal kinase (JNK) pathway as a homeostatic compensatory mechanism to replenish the apoptotic enterocytes. However, when *Pseudomonas aeruginosa* infected animals with a latent oncogenic form of the Ras1 oncogene, this homeostatic mechanism could lead to massive over-proliferation of intestinal cells (Apidianakis et al., 2009). The Imd–dTab2–dTak1 innate immune pathway was converged with Ras1V12 signaling on JNK pathway activation to induce the basal invasion and distant spread of drosophila’s posterior intestinal cells (Bangi et al., 2013). It means that bacterial infection could directly synergize with the genetic background to initiate stem cells-mediated tumorigenesis (Apidianakis et al., 2009; Bangi et al., 2013).

In addition to CRC, intestinal microbes have been involved in hepatic diseases due to the immunologic and metabolic communications between the liver and the intestine (Aykut et al., 2019). Yu et al. highlighted five potential key pathways that drove cancer-promoting liver inflammation, fibrosis, and genotoxicity, including leaky gut, microbe-associated molecular patterns (Toll-like receptor axis), dysbiosis, bacterial metabolites, and immunosuppression, which might synergize (Yu and Schwabe, 2017).

The tight connection between *H. pylori* and gastric cancer and the specific pathogenic mechanisms involving inflammation, immunity, and metabolism have been proposed (Goodwin, 1988). One well-known theory for the pathogenesis of *H. pylori* in gastric cancer is known as the leaking roof theory, which contains four potential steps that ultimately result in the barrier mechanism of the gastric mucosa being damaged. First, *H. pylori* penetrates the mucosal layer and settles on the surface of the gastric epithelial cells. Second, the bacteria releases toxic factors that damage the gastric epithelial cells. Third, various inflammatory cells and mediators appear. Fourth, the bacteria produce immunoreactive substances, in addition to others. The stepwise reaction might promote the gastric cancer (Goodwin, 1988). Further research is needed to confirm this theory. It was proposed that eradication of *H. pylori* infection could be used to prevent and cure gastric cancer (Tan and Wong, 2011; Lee Y. C. et al., 2016). In addition to *H. pylori*, lactic acid bacteria are thought to be associated with gastric cancer. Its pathogenic mechanism possibly includes the supply of exogenous lactate, which is a fuel source for cancer cells, promoting inﬂammation, angiogenesis, metastasis, epithelial-mesenchymal transition, immune evasion, production of ROS and N-nitroso compounds. Without the fuel, the survival rate of tumor cells would be greatly reduced. Lactic acid bacteria have anti-*H. pylori* properties that enable colonization by other non-*H. pylori* carcinogenic pathobionts (Vinasco et al., 2019).

To discover whether some other microbial mechanisms related to pancreatic cancer exist, besides changes in immune status (Pushalkar et al., 2018), Berk Aykut et al. observed that activation of the mannose-binding lectin–C3 cascade through the C3 complement pathway might cause inflammation induced by the oncogenic Kras, leading to fungal dysbiosis and promoting tumor progression. Inflammation might work as a consequence in metabolic progression, leading to cancer (Aykut et al., 2019).

## Conclusions

From the abovementioned polysystemic cancers, we summarized the diversity and distinguishing quantities of microbes, especially bacteria, in normal and tumoral tissues in most types of malignant tumors, which helped us promote the idea of an existing relationship between cancer and microbes (Nilsson et al., 2006; Guo et al., 2012; Kostic et al., 2012; Garrett, 2015; Mitsuhashi et al., 2015; Audirac-Chalifour et al., 2016; Lee Y. C. et al., 2016; Liu H. X. et al., 2018; Okushin et al., 2018; Aykut et al., 2019; Haruki et al., 2019). Analyzing microbial changes could indicate the etiopathology of disease, help in the design of novel diagnostic and treatment strategies, supervise and manage disease progression, and predict cancer prognoses (Liu H. X. et al., 2018). Bacteria have so far remained as the most studied microbes worldwide, and bacteria might affect the incidence and development of cancer through four major special pathogenic mechanisms, including modulating inflammation, immunity, DNA damage, and metabolism.

However, the formation of some cancers is not limited to only one pathogenic mechanism. Different pathogenic mechanisms might overlap in one cancer to induce tumorigenesis (Bouvard et al., 2009; Francescone et al., 2014; Kidane et al., 2014; Weyandt et al., 2017). Many exact microbial-driven pathogenic mechanisms of cancers have not yet been discovered. It is not known whether the role of the microbes is the etiology or the consequence (Christopoulos et al., 2014; Banerjee et al., 2017; Cavarretta et al., 2017; Khoruts, 2018; Pandey et al., 2019).Though many studies have considered microbes as the etiology of cancers, we cannot deny the possibility that microbial changes are an effect of some cancers. The specific tumoral environment of low immunity might provide a specialized niche to help microbes colonize (Christopoulos et al., 2014; Banerjee et al., 2017; Cavarretta et al., 2017; Khoruts, 2018; Pandey et al., 2019). More studies should be performed to confirm the real role of microbes. If the etiologic role and the precise pathogenic mechanisms of microbes in different cancers are determined clearly, the early prevention and treatment of cancers will greatly progress with a higher efficiency and accuracy.

## Author Contributions

QL, XF, and LH devised the conceptual idea. XF wrote the manuscript. LH, SM, LZ, LW, KZ, PY, LG, and WJ critically reviewed the manuscript. All authors contributed to the article and approved the submitted version.

## Conflict of Interest

The authors declare that the research was conducted in the absence of any commercial or financial relationships that could be construed as a potential conflict of interest.
